# Listening to the Fathers of Twins—Being Sensitive to Fathers’ Needs in Maternity and Child Healthcare

**DOI:** 10.3390/ijerph191710639

**Published:** 2022-08-26

**Authors:** Kristiina Heinonen

**Affiliations:** Faculty of Health Sciences, Department of Nursing Science, University of Eastern Finland, Yliopistonranta 1, 70210 Kuopio, Finland; kristiina.heinonen@uef.fi

**Keywords:** phenomenology, hermeneutic, van Manen, lifeworld, fatherhood, multiple-birth family, family care nursing, midwife, nurse

## Abstract

*Objective*: In a multiple-birth family, parenthood means being a parent to more than one child of the same age. The aim of this study was to describe the experiences of fathers of twins in order to contribute to the understanding of twin fatherhood and the needs for support. This article also provides some concrete guidance for midwives and nurses. *Design*: This qualitative research study was guided by the hermeneutic phenomenological approach. *Setting*: Notification of the study was published on the Multiple Births Association website for the fathers of twins. The data comprised fathers’ (n = 6) diaries and/or notes and in-depth interviews. *Results*: The following themes describe the phenomenon of being a father of twins: “Fatherhood of twins grows gradually”, “Strengthening of twin fatherhood by being present and involved”, “Father develops his relationship with each and both of the twins”, and “Making space for multiple fatherhood”. This article concentrates on the latter two themes. *Conclusions*: Fatherhood/parenthood is a very special time in a person’s life and has many effects on a child’s health and wellbeing and his/her life. Fathers of twins want to create a close bond with them by being actively present and involved in the children’s daily life, also with a view to the future. The staff of the hospital and maternity and child health clinic play a vital role in implementing services meant for multiple-birth families in the holistic understanding of and support for fathers/parents during the transition to parenthood and after the children’s birth. *Implications for practice:* Midwives and nurses are vital in providing support for, sharing knowledge with, and giving advice to fathers and different kinds of families. Multiprofessional cooperation that links evidence-based knowledge, theory, and practice, ensuring that the voices of both parents are heard and respected, is key to improving the care for different kinds of families and families with special needs.

## 1. Introduction

The birth of a child, becoming a parent, and parenting are special stages in a person’s life. Pregnancy and birth are transitional phases in the life of new parents [1]. Midwives and nurses who provide family nursing care come into contact with parents from different backgrounds, different cultures, and different family situations, such as two- and single-parent families, multiple-birth families, families with migrant backgrounds, bicultural families, and blended, adoptive, and rainbow families [2]. A multiple-birth family is one that is expecting multiples, or that has more than one child of the same age (i.e., twins, triplets, etc.). Three themes have been identified in fathers’ transition to parenthood: (1) emotional reactions: detachment, surprise, and confusion; (2) identifying their role as fathers: the approachable provider; (3) redefining the self and their relationship with their partner: a more united tag team [3]. New fathers’ role restrictions and changes in lifestyle have resulted in increased stress levels, which manifest also as tiredness, irritability, and frustration [4]. Although some studies found no differences, most investigations that compared the mental-health outcomes in parents of multiples versus parents of singletons found that the former experience heightened symptoms of depression, anxiety, and parenting stress, and may experience worse mental-health outcomes than the parents of singletons [5]. Despite increasing public awareness and sociopolitical changes that affect paternal parenting culture, fathers still seem to feel undervalued and unsupported in antenatal care (ANC) [6]. 

Having more than one child in a family not only brings a lot of joy, but also various challenges, and it makes it more difficult for parents to cope. Currently, the absolute and relative number of twins is at an all-time high globally [7]. Parents of multiples have highlighted the need for more support and information that are appropriate to their situation, and especially from maternity and child health clinics [8]. International research priorities for multiple-birth families include the promotion of parenting and family health, as well as the competence of professionals [9]. One of the ICOMBO objectives is to promote the principles detailed in the Declaration of Rights and Statement of Need of Twins and Higher Order Multiples [10].

Multiple pregnancies are always high-risk pregnancies, which require special monitoring and childbirth planning [11,12]. Twins are either monozygotic (MZ) or dizygotic (DZ) twins [13,14]. Twin pregnancy is associated with significant complications during and after pregnancy and childbirth, preterm birth, low weight, higher stillbirth rates, and the risk of neonatal and maternal death [7]. Awareness of the risks of multiple pregnancies increases parental anxiety and the need for support throughout pregnancy [8]. It is also stressful for the parent/family experiences [8,15,16,17] and fathers [18] when a child/children need(s) care in an NCIU or children’s ward. The lifeworld of multiple-birth parents can be described as a state of constant vigilance, ensuring that they can continue to cope, and having opportunities to share with other people [8].

Parents of multiples see parenting as a privilege and as something that increases happiness. A multiple birth is challenging, and parenting can be considered physically demanding [8,18,19]. Parents of multiples expect more information and support from maternity and child-health-clinic midwives and nurses for their special situation than they feel that they receive [8,19,20,21,22,23,24]. The meta-ethnography shows that fathers would like to receive more support and consideration for fatherhood [25]. Because of the baby care and other responsibilities with more than one child of the same age, fathers of multiples must adapt to fatherhood quickly [8,26,27]. Single parents of multiples need special consideration and support [23].

Establishing an early bond and interaction with both children can be difficult for parents of multiples [28]. A close relationship between the twins and being too reliant on each other affects the children’s health and wellbeing. Support is important in facilitating a mother’s ability to develop a relationship with each twin [8,29,30]. A difference in maternal attachment between twins and singletons has been found: the twins were more often Type B (secure/balanced) attached than the singletons, and on the basis of parental reports, the singletons had significantly more behavioral and emotional symptoms than the twins [31]. Parents of multiples feel the need for more information on how to interact with more than one child [24]. Family care workers working with multiple-birth families need special knowledge (e.g., on bringing up twins and treating them as individuals) [8,22]. The phenomenon of public health nurses supporting multiple-birth families can be expressed as “Recognising the strain”, “Lightening the load of daily life”, and “Targeting special needs” to the multiple-birth-family parents [24]. 

Care-related parental stress and shallow nocturnal sleep affect parental resilience. Finding a rhythm for only one of the twins, for example, has been found to make daily life easier and give parents the strength to cope [32,33]. Fathers were active in taking care of the infants during the night alone or sharing care, with each parent looking after one child. When the situation had settled and a rhythm had been found, the parents’ feeling that they could manage independently increased, strengthening the positive experience of parenthood [8]. Public health nurses have observed that insufficient sleep and sleep debt put a significant strain on parents’ resources, but they did not seem to have specific advice, information, or solutions for multiple-birth families in which two infants were calling and in need of attention [8,21,24].

Midwives and public health nurses working at clinics have brought up their lack of competence and need for training in issues related to multiple-birth families. There is a need for more education and understanding of the unique needs and life situations of such families [8,34]. Most health visitors had not received any specific training or continuing professional development regarding the needs of multiple-birth families [34]. Supporting the families within the confines of reduced time and an increased workload was challenging. The daily tasks of caring for multiples were the main areas that health visitors and parents wanted more information about [34].

Many fathers are interested in positive fathering, see themselves as much more than just passive supporters of their partners, and want to be genuinely engaged [8,35,36]. Fathers and public health nurses have described different ways of being a father, such as a bystander, supporter of a spouse, partner, and head of family [35]. Four different father types have been identified, depending on how they value fatherhood and how much they participate in the lives of their children: active and committed; active and weakly committed; passive and committed; and passive and weakly committed [37]. Together with parents, it is important to discuss how to establish a daily routine that takes account of the parents’ resources [23]. The essential meaning of the phenomenon of fathers’ expectations and experiences with municipal postnatal healthcare is described as going blindly into a women’s world, where fathers do not know what to ask, feeling excluded, seeking safety for the family, and longing for care [36].

Positive respectful behavior and language by nurses affected and supported men’s sense of involvement [38]. The meta-ethnography [25] shows that ANC still focuses more on the mother than the other parent, and that ANC should be developed to meet the different needs of mothers and other parents based on holistic understanding. Men were often not viewed or treated as equal partners during their transitions to fatherhood, and they lacked acknowledgment by or involvement with health professionals [4]. For some fathers, the sense of connection and involvement in the pregnancy increased as the pregnancy progressed, which appeared to be linked to men’s engagement in various activities during ANC [39]. However, some fathers also described feelings of being outsiders [40,41,42]. The fathers feel themselves to be a “partner and parent”, but they experience being “not-patient and not-visitor” in their encounters with maternity-care services [43]. Some fathers felt that social and healthcare professionals did not trust them as much as the mother in childcare, even though professionals find it important that fathers are also involved in ANC [8]. Fathers were welcomed but still passive, like strange visitors in a women’s world [36,44]. In a multiple-birth family, there are special needs during the pregnancy, and a need for help with caring for two newborn babies after birth and later. Being a father of twins has seldom been studied from the perspective of fathers alone, and this research gives the fathers of twins a voice to describe their experiences.

### The Aim

The aim of this study was to describe the experiences of fathers of twins in order to contribute to the understanding of twin fatherhood and the needs for support. This article also provides some concrete guidance for midwives and nurses.

## 2. Method

### 2.1. Epistemological Approach and Design

The hermeneutic phenomenological research approach and van Manen’s method were adopted in the current qualitative study. Merleau-Ponty’s four fundamental concepts of the lifeworld—spatiality, corporeality, temporality, and relationality, or communality—and van Manen’s materiality—are important in the research process of phenomenological questioning, reflection, and writing [17]. These existential themes are common to all human beings, regardless of their historical, cultural, or social situatedness, and they can be differentiated, but not separated. Nurses, doctors, and other health professionals need to be sensitive to the variety of ways that patient-families may experience their worlds [15,16]. 

### 2.2. Data Collection

A brief notification about the study was published on the website of the Multiple Births Association, stating that fathers of multiples were being sought for voluntary participation. The participants received more information about the study before giving their informed consent, and they were told that they could withdraw from the study without having to give a reason. Six (n = 6) fathers engaged in the process of submitting written descriptions and a discussion of these afterwards based on the father’s written diary/notes. The data were collected in 2018. The interviews allowed the fathers to say more about their experiences of fatherhood, referring to what they had written or what had come to their minds afterwards. The fathers ranged in age from 31 to 50 years, lived in different towns, and were professionals with different educational backgrounds working outside the home. The pairs of twins ranged in age from 2 to almost 9 years old.

### 2.3. Analysis

The research involved focused on the phenomenon, being interested in the experience as lived by individuals, reflecting on the essential themes, and describing the phenomenon while maintaining a strong relation to it and balancing the research context. The holistic approach depended on understanding the research data and the experiences of fathers of twins. In the selective and detailed phase, the researcher chose illuminating quotes from what the fathers wrote or said and rewrote every experience of each participant. The researcher combined the unique themes remembering the individual experiences and turned the phenomenon of being a father of twins into a disciplinary understanding [17,45].

## 3. Results

This article presents two themes from the phenomenon of being a father of twins: “Father develops his relationship with each and both of the twins” and “Making space for multiple fatherhood”.

### 3.1. Father Develops His Relationship with Each and Both Twins

#### 3.1.1. The Joy of Fatherhood and Having Two Children of the Same Age

Fatherhood is an active and wide-ranging involvement in caring, and it follows twin growth and development. The father can observe various aspects of each child’s growth and development. Fatherhood is the joy of each and both twins, and the shared moments between a father and twins, both together and singly, such as watching them play. It is a great moment when the father sees that his twins missed him, and when he feels that he is important in their life:

“The first time the kids spontaneously hugged each other was quite moving. The first shared games, chatter and babble… all that is almost magical… The children couldn’t even walk yet when one vigorously pushed the other twin forward on the Bobby Car…”

“… it’s a wonderful reception on a weekday when two children are waiting for you and run to you when you come home from work. Oh, that joy and happiness… the joy of seeing daddy again…”

#### 3.1.2. Building and Nurturing the Relationship with Each and Both of the Twins

The fathers considered it important to form an equal bond with both twins also by being with and giving time to each child separately. The relationship between a father and twin(s) is one of the most important aspects of fatherhood, but the question of when it was a good time to be alone with one child was confusing for the fathers. They revealed that they had also pondered how to engage with the twins equally:

“… I don’t see that in any way one would come first, indeed they are absolutely equal to me…”

“… different to be with just one twin in the car… we chatted… somehow a different dynamic. However, I would love to get to know the child more, to bring out the individuality of both twins separately… I can’t say when it would be time, and whether it will do any good or not …”

“… it would be important for me as the father to be able to give even more time to one child. It helps me see that child as a separate individual, because of course they’re not the same person. Just for practical reasons, however, we’re often together and then I sometimes speak to them in turn…”

One father thought that he sometimes gave more attention to the male child and got on better with him. It was not a question of favoring the male twin, but rather that he felt that he and the boy were more alike, and he identified with the boy because he was once a little boy himself.

#### 3.1.3. The Trials of Being a Father of Twins

Fatherhood changes over time as the children grow and develop, and it is tested by various emotions, including conflicting ones, the demands of fatherhood, and the lack of time for his relationship with the mother. Fatherhood is also tested by the children’s developmental issues:

“… everyday life is sometimes a test as a father on childcare leave and I try to get one twin’s nappy changed and then the other one goes and makes everything impossible or when food is constantly thrown on the floor during meals… then that’s the hardest time.”

### 3.2. Making Space for Multiple Fatherhood

#### 3.2.1. Strengthening Twin Fatherhood

The resources for fatherhood come from joy, the twins, fostering his relationship with the mother, and opportunities for time of his own. A father-positive approach by social and healthcare professionals is important. Some of the fathers had also encountered surprise and suspicion about their being so actively present in family life:

“Being with the twins has helped me grow more into this fatherhood all the time. My fatherhood has been strengthened by being able to be present with the twins and in everything. However, it has been a bit puzzling to hear surprise expressed from many quarters that I also take care of them alone…”

The fathers expected more attention and support for the fatherhood of twins from social and healthcare professionals:

“The clinic provides quite understandable information, but it tends to be directed at the mother. As a father, you stay in the background if you are not active yourself and don’t ask questions. Fatherhood should be brought more to the fore and taken into account. Getting information surely can’t be clinic specific.”

“Fatherhood is definitely left to you yourself and your own family… it’s something that could be discussed at the clinic…”

#### 3.2.2. Making Time for Yourself and Your Own Twin Fatherhood

Being a father requires time of his own and for his own things. Fatherhood involves thinking about his relationship with his own father and his own childhood. Time is also required for the relationship between the father, twins, and mother. In traveling the same path with the twins, fathers should have a positive attitude towards being a father of twins:

“… it would be good if we could sometimes, perhaps… be together, mother with one of the twins, say, and father with the other…”

“… I have occasionally already started going back to my old hobbies and groups of male friends. We exchange news and talk about our families, too… and our children. Nice to share things and my own fatherhood. It really is so unique in this life.”

## 4. Discussion

The essence of the phenomenon of being a father of twins is that fatherhood of twins grows gradually, with the father being present and involved as he develops his relationship with each and both of the twins. Fatherhood brings enormous joy to the father from his own children, as well as responsibility and trials. Being a father of twins is a complex process that develops from the pregnancy onwards. Fatherhood means being actively present and taking care of the twins; it awakens and becomes more concrete after their birth. Memorable moments with the twins promote the fatherhood process, are emotionally powerful, and encompass a variety of feelings. Fatherhood also involves an instinctive need to protect the twin babies. At maternity and childcare welfare clinics, midwives and nurses meet parents only according to the need and the guidelines on monitoring the pregnancy and the children’s growth and development. This is an important time for parents, and it is vital that midwives show interest and support parenthood. Unfortunately, parents may not always see the same midwife/nurse, which hinders the continuity of care. It is also important to ask for feedback from parents and develop the quality of family nursing care. Fathers of multiples must adapt to fatherhood quickly because of the extra baby care [8,26,27], and they need comprehensive and special information on things such as taking care of twins and promoting individuality [8,22,24]. There is a need for more guidance and support regarding the preparation for fatherhood and relationship changes with their partner [35,36,37]. 

Professionals need to understand the lifeworld of fathers/other parents of twins. This enables them to promote and support such parenthood. When the midwife has a close professional relationship with the father/other parent as well, she can help him grow into parenthood and find his role as a parent. Fathers/other parents have resources that also help mothers cope with the challenges of multiple parenthood, and to share the burden of childcare and household tasks. The present study shows that the fathers experienced strong emotions and a feeling of oneness with their children. They described moments with the children that were different and special compared with ordinary daily life, and they described how they felt that such moments strengthened their fatherhood, as well as the father–child bond. Active fatherhood promotes the interaction and attachment between parents and twin/twins [31]. Midwives can support this process by asking them to talk about their feelings and experiences while making positive comments, asking questions, and giving them room to say more. These are also important moments to help fathers recognize each child as an individual, and to talk about their observations. This study also shows that fathers of twins would like to be more visible and fully recognized as parents at the maternity and child-welfare clinic. Midwives and nurses should initiate an open discussion with fathers/partners about how they see and experience their own fatherhood/parenthood, and how they can strengthen it (Table 1).

The fathers said that they saw each child differently, and that the atmosphere was different when they were alone with one child, compared with the situation when both twins were present, although both situations are important to the fathers. Parents need information and support in seeing the twins as individuals and in giving them individual attention. As part of this process, parents should be advised to devote enough time to each twin separately, talking to and maintaining eye contact with the individual. Such sessions complement the time spent with both twins together. Parents of twins need special knowledge about intertwin relationships. There is dominance–submissiveness between co-twins from three separate perspectives: physical, psychological, and verbal [17,45]. Ref. [31] notes that there were no significant differences between the distributions of attachment to the mother and father when both parents took care of the twins. The intertwin relationship and the company of others may also compensate for the lack of maternal time and attention [31]. In middle adolescence, puberty appeared to be the most difficult phase for twins; some exhibited depressive and psychosomatic/somatic symptoms. Mutual dependence influences twins’ social interactions, leisure activities, and later, their psychiatric and psychosomatic wellbeing [30]. This dependence was deeper in female monozygotic (MZ) twins [29].

Sometimes fathers need to be encouraged to spend time alone with the children. Problem solving is one way to find solutions beforehand and use them when needed. For example, preparing for a situation where one of the twins is in the bath with the father and the other child starts crying. Singing or having peaceful music or a babysitter can be helpful when taking care of the children alone, or sleeping in different rooms, with each parent taking care of one child. One solution is to “share the twins”: a mother’s twin and a father’s twin [8,46,47]. This solution has been found to promote the individual development of twins and to reduce the mutual rivalry between them, as each has his/her own parent as an object of identification and attachment [46]. Parents find it important to change children and to become acquainted with both/all of them, which deepens the parent–child relationship and interaction [8]. The father’s participation in childcare reduces the competition between the twins. Twins who did not feel that they were clearly the children of the mother or of the father suffered the least from psychosomatic symptoms [30,46]. In the current study, fathers see themselves mostly as active and committed, giving time to their children, and as the head of the family, with a participating and caring role. This is a subject that midwives can discuss with fathers, and they should also consider how to encourage fathers who are not strongly committed to fatherhood. Perhaps emotional moments with the twins could help them to understand the importance of interacting with each and both twins and observing their behavior and reactions. Fathers would like to have specific information and advice that is targeted to multiple-birth families, and to have their voices heard by midwives and nurses. Public health nurses tried to gain an understanding of the daily life and need for support of multiple-birth families by being present, listening to parents, showing empathy, and giving advice. Peer support compensates for the evidence-based support provided by midwives and nurses [8,22].

Social and healthcare education and professional competence should be improved by using evidence-based knowledge and support for parenthood. Midwives and nurses may be able to help parents by using the salutogenic model [48,49]. This might assist in promoting the transition to parenthood by supporting the sense of coherence. Promoting comprehensibility may help with orienting parents to their new situation, while manageability involves organizing life around the children, discussion, preparation, and sharing with the other parent, and meaningfulness relates to giving yourself time to grow into parenthood. Together with the concept of the lifeworld, this could provide a universal basis for midwives and nurses to support parents and the whole family while promoting their health and wellbeing. It is important to broaden the understanding of multiple-birth families, provide information, guidance, and advocacy, and build connections with multiples groups worldwide, as well as with other organizations and professionals that work in different areas of childcare [50]. Multiple-birth families have their special needs in family nursing care. Many studies state that social and healthcare professionals require more knowledge, experience, and training to support multiple-birth families, given the vital role that they play in supporting and helping the parents of multiples [8,20,22,28,34]. Multiprofessional cooperation that links research, theory, and practice, ensuring that the voices of parents are heard and respected, is key to improving the care for multiple-birth families. There is a need for multiprofessional wide-ranging cooperation and determination. 

### 4.1. Ethical Considerations

The Helsinki Declaration principles were followed during the research process [51]. The assessment of the research and data-protection issues was performed by the Norwegian Centre for Research Data (NSD), and a favorable opinion was obtained from the Ethics Committee (NSD 2018/60447). Participants were given time to consider the matter before they signed and could withdraw from the study at any time. The participants’ autonomy was vital and ensured throughout the research process. Only the researcher interviewed the participants and knows which data belong to which participant. Because of the small number of participants, the direct quotations are without numbers.

### 4.2. Strengths and Limitations

Phenomenological hermeneutic studies respect natural enquiry into real contexts. The main point is to open discussion while respecting the uniqueness of the experiences of fathers of twins. Evidence-based knowledge is important to understand people in all their variety. The outcome of the study should contain just the right amount of experiential material that creates a scholarly and reflective phenomenological text. In the current study, heuristic questioning means wondering about the phenomenon of being a father of twins throughout the research process, with the descriptive richness involved giving the fathers the opportunity to write about their experiences, and afterwards, to open them up orally. In memory and lived time, being a father to more than one child of the same age is presented as a lived experience, and the description, converted into an anecdote, was subjected to holistic, selective, and line-by-line thematizations. The analysis generated themes for this distinct meaning of the phenomenon (distinctive rigor) [17,45]. The fathers found it good to be interviewed by phone, which supported their anonymity in openly talking about their personal and sensitive experiences. There is a lack of comparability of the study, as this field is relatively unexplored. This also highlights the importance of the current study [33]. The author has experience in this kind of research, and in multiple-birth-family research, and has a background as a public health nurse.

## 5. Conclusions

Fatherhood/parenthood is a very special time in a person’s life, and it has many effects on a child’s health and wellbeing and life. Fathers of twins consider fatherhood to be a very important time and want to make the most of it. Fathers of twins want to create a close bond with them by being actively present and involved in the children’s daily life, also with a view to the future. Providing support for fathers/partners also helps to promote the health and wellbeing of the whole family. The involvement of fathers/other parents should be strengthened in the social and healthcare services. The staff of the hospital and maternity and child health clinic play a vital role in implementing services meant for multiple-birth families, and other families with special needs, by supporting fathers/parents during the transition to parenthood, and after the children’s birth.

## Figures and Tables

**Table 1:** Tips for midwives and nurses to support the fathers of twins in their parenthood.

Theme	Support for the Father/Other Parent
**Father develops his relationship with each and both of the twins**	
Building and nurturing the relationship with each and both twins	Emphasize the importance of fatherhood for the father himself, the children, and the whole family Discuss responsible parenthood Strengthen and support fatherhood/parenthood Encourage him to spend time with the children also without the other parent Give the father confidence that he can cope Encourage him to give time to each child when the twins are together, but also without the other twin Advise him to make time to be with both twins together and with each separately Listen to the father when he tells you about these moments, ask questions Give positive feedback Ask him to talk about and share his experiences of fatherhood and the emotions it arouses Ask him to tell you about his observations regarding the children/each child Encourage him to recognize the individuality of each child
The joy of fatherhood and of having two children of the same age	Encourage him to talk about and share with you what it is like to be a father of multiples Encourage him to accept different emotions associated with fatherhood Listen to and support the father’s observations concerning the individuality of each twin/the twins Help him to see the children as individuals Help him to see the specialness of a multiple-birth family
The trials of being a father of twins	Discuss matters related to each child’s growth and development Tell him that fatherhood also involves difficult situations and that such moments will help him grow into parenthood Tell him that situations are different with more than one child of the same age Bring up the topic of parental love for each child/the children Help him acknowledge and congratulate himself when he has coped with and made it through a situation that sapped his resources Help him focus on the good moments Advise him to discuss and decide things together with the other parent so that they do things in a similar way Guide him to moments and things that will boost his resources Guide him to things in daily life that will boost his resources
**Making space for multiple fatherhood**	
Strengthening twin fatherhood	Encourage him to be active when visiting the maternity and child welfare clinic Strengthen his involvement Encourage him to boldly ask questions about fatherhood/parenthood Encourage and support discussion Give time and space for fatherhood
Making time for yourself and your own twin fatherhood	With the consent of the fathers/parents, try to bring families in the same life situation together for peer-support family training and also ask for the father’s/other parent’s views Give fathers the opportunity to meet each other in their own group for peer support Encourage parents to foster a feeling of togetherness and make time for brief moments together in their daily life Encourage the father to pay attention to the couple’s relationship in their daily life Encourage him to enjoy fatherhood in the here and now

## References

[1] Meleis A.I. (1997). Theoretical Nursing: Development End Progress.

[2] The Family Federation of Finland (2021). Terveyden Ja Hyvinvoinnin Laitos. https://thl.fi/fi/web/sukupuolten-tasa-arvo/tasa-arvon-tila/perheet-ja-vanhemmuus/perheiden-moninaisuus.

[3] Chin R., Hall P., Daiches A. (2011). Fathers’ experiences of their transition to fatherhood: A metasynthesis. J. Reprod. Infant Psychol..

[4] Baldwin S., Malonel M., Sandall J., Bicks D. (2018). Mental health and wellbeing during the transition to fatherhood: A systematic review of first time fathers’ experiences. JBI Database System Rev. Implement. Rep..

[5] Wenzeler S.J., Battle C.L., Tezanos K.M. (2015). Raising multiples: Mental health of mothers and fathers in early parenthood. Arch. Womens Ment. Health.

[6] Kowlessar O., Fox J.R., Wittkowski A. (2015). First-time fathers’ experiences of parenting during the first year. J. Reprod. Infant Psychol..

[7] Monden C., Pison G., Smits J. (2021). Twin Peaks: More twinning in humans than ever before. Hum. Reprod..

[8] Heinonen K. (2013). The Lifeworld of Multiple-Birth Families from Being on Guard to Strengthening Parenthood. Phenomenological-Hermeneutic Study. Ph.D. Thesis.

[9] Lam J.R., Liu B., Bhate R., Fenwick N., Reed K., Duffy J.M.N., Kahlil A., Global Twins and Multiples Priority Setting Partnership Collaborators (Heinonen, K.) (2019). Research priorities for the future health of multiples and their families: The Global Twins and Multiples Priority Setting Partnership. Ultrasound Obstet. Gynecol..

[10] International Council of Multiple Birth Organisations (2022). Strategic Plan 2022–2024: Championing the Rights of Multiples.

[11] Klementti R., Hakulinen-Viitanen T. Äitiysneuvolaopas. Suosituksia Äitiysneuvolatoimintaan. Kirjoittanut Kansallinen Asiantuntijatyöryhmä. Äitiysneuvolan Tavoitteet ja Toimintaa Ohjaavat Periaatteet. THL, 2013. https://www.julkari.fi/bitstream/handle/10024/110521/THL_OPA2013_029_verkko.pdf?sequence=3&isAllowed=y.

[12] Tiitinen A. (2022). Monisikiöisyys (Monikkoraskaus).

[13] Purho J., Nuutila M., Heikinheimo O. (2008). Kaksosraskaudet. Duodecim.

[14] Kaprio J., Toimittaneet Kumpula U., Kaprio J., Lavikainen A., Moilanen I. (2020). Monikkoraskaudet ja kaksosmalli. Kiehtova Kaksosuus. Monikkosisarusten Elämää.

[15] Van Manen M.A. (2014). On ethical (in)decisions experienced by parents of infants in neonatal intensive care. Qual. Health Res..

[16] Van Manen M.A. (2019). Phenomenology of the Newborn: Life from Womb to World.

[17] Van Manen M. (2014). Phenomenology of practice. Meaning-Giving Methods in Phenomenological Research and Writing.

[18] Renfors R., Kaunonen M., Koivisto A.-M. (2019). Isien stressi vastasyntyneiden teho- ja tarkkailuosastolla. Hoitotiede.

[19] Beck C.T. (2002). Mothering Multiples. A methasynthesis of qualitative research. MCN Am. J. Matern. Child Nurs..

[20] Harvey M.E., Athi R., Denny E. (2014). Exploratory study on meeting the health and social care needs of mothering with twins. Community Pract..

[21] Heinonen K. (2015). Methodological and hermeneutic reduction—A study of Finnish multiple-birth families. Nurse Res..

[22] Heinonen K. (2016). Supporting Multiple Birth Families at Home. Int. J. Caring Sci..

[23] Heinonen K. (2019). Describing Being a Single Parent of Multiples. Int. J. Caring Sci..

[24] Heinonen K., Häggman-Laitila A., Moilanen I., Pietilä A.-M. (2019). The Lifeworld of Multiple Birth Families. Int. J. Caring Sci..

[25] Heinonen K. (2021). Strengthening Antenatal Care Towards a Salutogenic Approach: A Meta-Ethnography. Int. J. Environ. Res. Public Health.

[26] Leonard L.G., Denton J. (2006). Preparation for parenting multiple birth children. Early Hum. Dev..

[27] Huttunen J., Kumpula U., Kaprio J., Lavikainen A., Moilanen I. (2020). Kerralla monen vanhemmaksi. Kiehtova Kaksosuus. Monikkosisarusten Elämää.

[28] Bryan E. (2008). Multiple birth Children and their families—What nurses need to know. AWHONN Linelines.

[29] Penninkilampi-Kerola V. (2006). Implications of Co-Twin Dependence for Twins’ Social Interactions, Mental Health and Alcohol Use: A Follow-up Study of Finnish Twina from Adolescence to Early Adulthood. Ph.D. Thesis.

[30] Trias T. (2006). Inter-Twin and Parent-Twin Relationships and Mental Health. A Study of Twins from Adolescence to Young Adulthood. Ph.D. Thesis.

[31] Tirkkonen T., Kunelius A., Ebeling H., Pylkkönen M., Tuomikoski H., Moilanen I. (2008). Attacment in Finnish singletons and twins both parents. Psychiatr. Fenn..

[32] Thome M., Skuladottir A. (2005). Evaluating a family-centered intervention for infant sleep problems. J. Adv. Nurs..

[33] Damato E.G., Burant C. (2008). Sleep patterns and fatigue in parents of twins. J. Obstet. Gynecol. Neonatal. Nurs..

[34] Turville N., Alamad L., Denton J., Cook R., Harvey M. (2021). Supporting multiple birth families: Perceptions and experiences of health visitors. Public Health Nurs..

[35] Kaila-Behm A., Vehviläinen-Julkunen K. (2000). Ways of being a father: How first-time fathers and public health nurses perceive men as fathers. Int. J. Nurs. Stud..

[36] Hogmo B.K., Bondas T.E., Alstveit M. (2020). Going blindly into the women’s world: A reflective lifeworld research study of fathers’ expectations of and experiences with municipal postnatal healthcare services. Int. J. Qual. Stud. Health Well-Being.

[37] Huttunen J. (2001). Isänä Olemisen uudet Suunnat. Hoiva-isiä, Etä-isiä ja Ero-isiä.

[38] Johansson M., Fenwick R.M., Premberg R.N. (2015). A meta-synthesis of fathers’ experiences of their partner’s labour and the birth of their baby. Midwifery.

[39] Wilmore M., Rodger D., Humphreys S., Clinton V.L., Dalton J., Flabouris M., Skuse A. (2011). How midwives tailor health information used in antenatal care. Midwifery.

[40] Premberg A., Hellström A.-L., Berg M. (2008). Experiences of the first year as a father. Scand. J. Caring Sci..

[41] Draper J. (2002). It’s the first scientific evidence: Men’s experiences of pregnancy confirmation. J. Adv. Nurs..

[42] Draper J. (2003). Men’s passage to fatherhood: An analysis of the contemporary relevance of transition theory. Nurs. Inq..

[43] Steen M., Downe S., Bamford N., Edozien L. (2012). Not patient and not-visitor: A meta-synthesis of fathers’ encounters with pregnancy, birth and maternity care. Midwifery.

[44] Olsson P., Sandman P.O., Jansson L. (1996). Antenatal “booking” interviews at midwifery clinics in Sweden: A qualitative analysis of five video-recorded interviews. Midwifery.

[45] Van Manen M. (1997). Researching lived experience. Human Science for an Action Sensitive Pedagogy.

[46] Moilanen I., Pennanen P. (1997). “Mother s child” and “father s child”among twins. A lognitudial twin study from pregnancy to 21 years age, with special reference to development and psychiatric diaorders. Acta Genet. Med. Gemellol..

[47] Moilanen I., Kunelius A., Tirkkonen T., McKinsey Grittenden P., Crittenden P.M., McKinsey P., Claussen A.H. (2003). Attachment in Finnish twins. The Organization of Attachment Relationships: Maturation, Culture and Context.

[48] Antonovsky A. (1979). Health, Stress, and Coping.

[49] Antonovsky A. (1987). Unraveling the Mystery of Health: How People Manage Stress and Stay Well.

[50] ICOMBO (2021). International Council of Multiple-Birth Organisations. http://icombo.org/.

[51] World Medical Association (2013). World Medical Association Declaration of Helsinki: Ethical principles for medical research involving human subjects. J. Am. Coll. Dent..

